# Nationwide prospective cohort study in China: the impact of cumulative modified cardiometabolic index on cardiovascular disease incidence

**DOI:** 10.3389/fcvm.2026.1836056

**Published:** 2026-06-05

**Authors:** De-Heng Pu, Li-Lin Zhang, Jing Yang, Xian-Bin Wang, Hao Luo

**Affiliations:** 1 Department of General Medicine, Ya'an People’s Hospital, Ya'an, Sichuan, China; 2 Department of Orthopaedics, Xiangya Hospital, Central South University, Changsha, China; 3 National Clinical Research Center for Geriatric Disorders, Xiangya Hospital, Central South University, Changsha, Hunan, China

**Keywords:** CHARLS, cumulative exposure, cvd, linear dose-response relationship, MCMI, prospective cohort study

## Abstract

**Background:**

The global burden of cardiovascular disease (CVD) is rising. The modified cardiometabolic index (MCMI), which includes dyslipidemia, impaired glucose tolerance, and central obesity, effectively predicts metabolic diseases. There are no studies examining whether cumulative MCMI (cumMCMI) exposure is associated with the onset and progression of CVD. This research aims to determine if increased cumMCMI correlates with higher CVD rates.

**Methods:**

The first three waves of the “China Health and Retirement Longitudinal Study” (CHARLS) excluded individuals with CVD, missing MCMI data, or not meeting study criteria, resulting in 3,466 eligible participants. We assessed the impact of high cumMCMI on CVD risk over a median 5-year follow-up using multivariate Cox regression and Kaplan–Meier curves, considering exposure duration and analyzing both continuous variables and quartiles. The dose-response relationship of cumMCMI was evaluated using restricted cubic splines (RCS), while subgroup analyses examined population characteristics. Researchers assessed model robustness by varying exposure thresholds and employing Poisson and logistic regression models.

**Results:**

In all models, the Q4 group showed the highest hazard ratio (HR) [HR 1.51, 95% confidence interval (95%CI) 1.13–2.00, *P* = 0.005], indicating that cumMCMI is a CVD risk factor. RCS analysis indicates the presence of a linear positive correlation in CHARLS (*P* for overall = 0.015, *P* for nonlinearity = 0.737). Subgroup analyses found higher risks for those with lower education and married individuals (*P* for interaction < 0.05). Sensitivity analyses yielded materially similar results.

**Conclusion:**

In short, cumMCMI is independently linked to CVD and could aid in CVD stratification and early warning. Although education and marital status might influence this connection, further validation is needed.

## GRAPHICAL ABSTRACT

**Figure:**
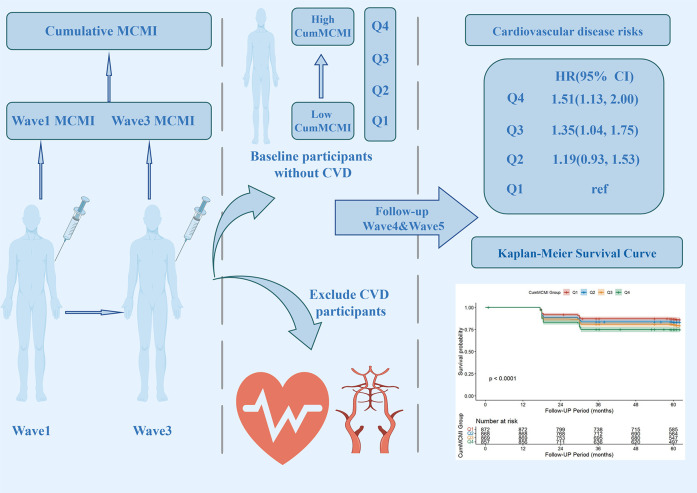
cumMCMI, Quartiles: Q1 (cumMCMI < 7.38), Q2 (7.38 ≤ cumMCMI < 8.62), Q3 (8.62 ≤ cumMCMI < 9.97), Q4 (9.97 ≤ cumMCMI). MCMI, modified cardiometabolic index; cumMCMI, cumulative modified cardiometabolic index; CVD, cardiovascular disease (this graphical abstract was created with the “home-for-researchers” membership feature and is authorized by the platform). created with figdraw.com.

## Introduction

Since 1990, the global prevalence of CVD has more than doubled, rising by 101.3% from 311 million to 626 million cases by 2023. During the same period, global deaths from CVD increased by 46.6%, from 13.1 to 19.2 million (1). Dyslipidemia (2, 3), impaired glucose tolerance (3, 4), and central obesity (3, 5, 6) are crucial in the development and advancement of CVD. Traditional CVD risk assessments frequently depend on individual, clinically accessible indicators, such as low-density lipoprotein cholesterol (LDL-C) (7), waist-to-height ratio (8, 9), fasting blood glucose (FBG) (10), high-density lipoprotein cholesterol (HDL-C) (11), or triglycerides (TG) (12). Nonetheless, the pathogenic effects associated with these indicators are generally marked by substantial long-term cumulative impacts (13). Thus, accurately measuring long-term exposure to various metabolic risk factors, including blood test outcomes and body measurements, is vital for detecting CVD early in the general populace.

Historically, extensively researched metabolic indices have predominantly concentrated on dyslipidemia, impaired glucose tolerance, and obesity, analyzing their correlations with chronic diseases. Nonetheless, these indices exhibit varying degrees of potential for further enhancement. For instance, the triglyceride-glucose (TyG) index incorporates TG and FBG but omits a measure for central obesity. Similarly, the atherogenic index of plasma (AIP) considers only TG and HDL-C, while the cardiometabolic index (CMI) does not account for abnormalities in FBG levels, leading researchers to propose the MCMI, which includes FBG levels and is linked to metabolic disease (14). Recent research has verified that higher baseline MCMI levels are linked to a greater likelihood of chronic illnesses like CVD. Recent research comparing the MCMI and TyG indices in predicting the incidence of type 2 diabetes (T2DM) indicates that the MCMI exhibits superior predictive capability (15). Several recent CHARLS-based studies have evaluated baseline MCMI in relation to chronic disease outcomes, cardiometabolic multimorbidity, or CVD risk in specific high-risk populations. However, these studies primarily assessed MCMI as a single-timepoint exposure and did not address whether long-term cumMCMI exposure is associated with future CVD risk in a community-based middle-aged and older population. Therefore, constructing cumMCMI from repeated biomarker measurements may provide additional insight into the role of sustained cardiometabolic burden in CVD development (14, 16–18).

This study aims to create a cumMCMI from the CHARLS and analyze its distribution, the dose-response relationship with new-onset CVD, and variations across age, gender, and baseline health status.

## Materials and methods

### Research design and data

Participants in this study were chosen from the CHARLS cohort, which began in 2011–2012 (Wave 1). Follow-up data has been collected in four waves: 2013 (Wave 2), 2015 (Wave 3), 2018 (Wave 4), and 2020 (Wave 5). Blood samples were only collected during the first and third waves. For more information on the study's rationale, design, and sample collection, refer to previous publications (19, 20). The USC Global Aging Data Portal team created the “Harmonized CHARLS”. For more information on these datasets, including questionnaires and metadata, visit https://g2aging.org/. Since Wave 5 and blood sample data are absent from the Harmonized CHARLS dataset, we processed our data based on the Harmonized CHARLS dataset and merged the Wave 5 and blood sample data using anonymized participant IDs.

**Figure 1:**
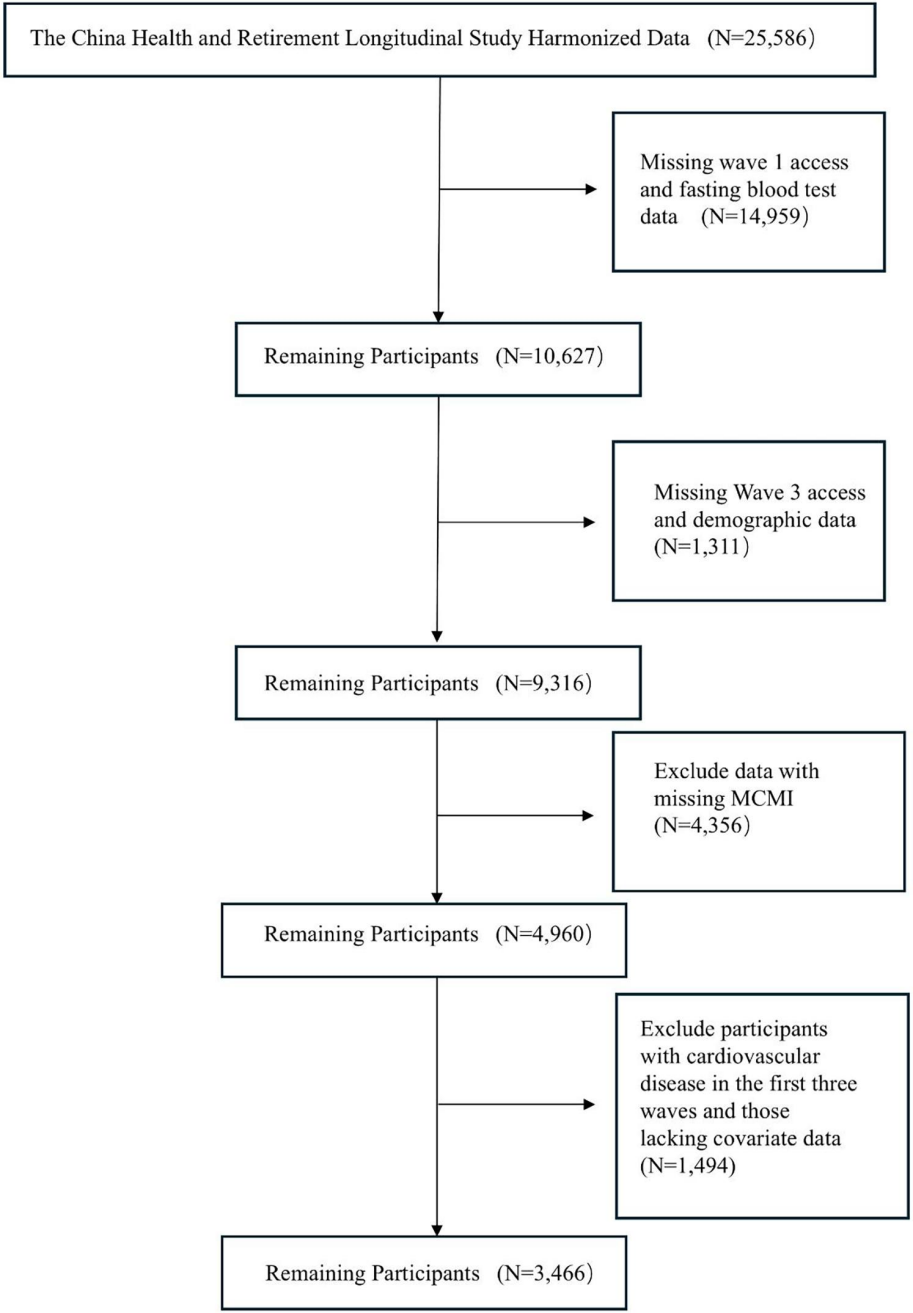
Flowchart of study population selection for the study. MCMI, modified cardiometabolic index.

Using the Harmonized CHARLS dataset (*N* = 25,586), we combined data from various sources: Wave 1 and Wave 3 blood samples, the Wave 5 Health Status and Functioning questionnaire, and the Wave 5 exit questionnaire. This formed the basis of our study. We excluded participants with incomplete data from Wave 1 interviews or fasting blood tests (*N* = 14,959), missing data from Wave 3 interviews or demographics (*N* = 1,311), and those without MCMI data (*N* = 4,356). After also excluding individuals with CVD in the first three waves and those lacking in necessary covariate data (*N* = 1,494), the final cohort included 3,466 participants (Figure 1).

Each wave of the CHARLS survey received authorization from the Biomedical Ethics Committee at Peking University. This study used only anonymized and de-identified data from CHARLS, without any effort to re-identify individuals or merge data with other sensitive information, as per the ethical exemption in the usage agreement.

### Determining CVD outcomes

The study's principal outcome is the self-reported physician diagnosis of heart disease or stroke, which indicates the presence of CVD (21).

### cumMCMI calculation

This formula is used to determine the MCMI (14):

$$MCMI=\ln\left(\frac{FBG(\mathrm{mg}/\mathrm{dL})\times TG(\mathrm{mg}/\mathrm{dL})}{HDL-C(\mathrm{mg}/\mathrm{dL})}\right)\times \frac{WC(\mathrm{cm})}{Height(\mathrm{cm})}$$

$$cumMCMI=(MCMI_{wave1}+MCMI_{wave3})÷2\times Time(2015-2012)$$

### Covariates

The study considered age, sex, marital status, education, residence, LDL-C, hemoglobin A1C (HbA1c), TC, smoking status, drinking status, liver disease, diabetes, hypertension, kidney disease, dyslipidemia, overweight, depression, antihypertensive drug, antidiabetic drug, and lipid-lowering drug as covariates. The method for identifying conditions like hypertension aligns with the previously described method for assessing CVD. According to CHARLS, a body mass index (BMI) over 23.9 kg/m2 indicates that they are overweight. CHARLS incorporates the 10-item Center for Epidemiological Studies Depression Scale (CES-D). According to prior research, a CES-D score of 10 or above is indicative of clinical depression (22).

### Statistical analysis

Continuous variables adhering to a normal distribution are indicated as mean ± SD, whereas those that do not are indicated as the median (IQR). Percentages (%) were the format used for categorical variables. For variables with skewed distributions, the Kruskal–Wallis test is used. Chi-square tests facilitated the comparison of categorical variables. To compute the HR and 95% CI for CVD, we applied a Cox regression model with adjustments for multiple variables. This research initially looks at cumMCMI as a continuous variable, and then further investigates it as a categorical variable categorized by quartiles (Q1 (cumMCMI < 7.38), Q2 (7.38 ≤ cumMCMI < 8.62), Q3 (8.62 ≤ cumMCMI < 9.97), Q4 (9.97 ≤ cumMCMI)). Four regression models were used: Crude Model served as the baseline without adjustments; gender, age, residence, education, and marital status were included in Model 1 for adjustment; Model 2 included further adjustments for smoking status, drinking status, LDL-C, TC, HbA1C; and diabetes, hypertension, hyperlipidemia, kidney disease, liver disease, overweight, depression, antihypertensive drug, antidiabetic drug and lipid-lowering drug are further adjusted for in Model 3. The Kaplan–Meier curve estimated CVD prevalence, and the log-rank test assessed differences between curves.

An RCS model was introduced to check for the relationship between CVD and cumMCMI. All analyses were adjusted for covariates in Model 3. To further pinpoint potential at-risk groups, a subgroup analysis was carried out. Each stratum was adjusted for Model 3 variables except for the stratification factor.

We performed three sensitivity analyses to test result robustness: (1) Participants were split into high- and low-exposure groups using the median cumMCMI as a cutoff, applying the inverse probability-weighted Poisson regression; (2) The exposure cutoff was changed to the upper quartile, using the same model; (3) The *E*-value was calculated via a weighted logistic regression model. A *P* value of less than 0.05 in a two-tailed test demonstrated statistical significance. Using R (version 4.4.1), all analyses were carried out.

## Results

### Demographic information

The analysis involved 3,466 participants without CVD, median age 57, including 1,880 women and 1,586 men. The initial characteristics of cumMCMI quartiles are shown in Table 1. Significant differences were found in gender, age, residence, smoking status, diabetes, drinking status, dyslipidemia, hypertension, overweight, antihypertensive drug, antidiabetic drug, lipid-lowering drug, HbA1C, LDL-C, TC, cumMCMI, follow-up time, and CVD incidence across groups (*P* < 0.05), while marital status, education, liver disease, and kidney disease distributions were similar. Men made up 64% of the Q1, while women comprised 70% of the Q4. Rural residents were most prevalent in Q1 (91%), but urban residents increased from 8.8% to 16% as cumMCMI rose. The median age was 56–58 years across groups, with Q1 slightly older at 58 years. As cumMCMI levels increased, the proportions of current smokers and drinkers decreased, while never-smokers, never-drinkers, and those without these conditions increased. From Q1 to Q4, LDL-C, TC, HbA1C, and cumMCMI increased, while the prevalence of hypertension, dyslipidemia, diabetes, overweight, use of related medications, and incidence of CVD also rose. The Q4 lower quartile follow-up time was shorter than other groups (Table 1).

**Table 1:** Baseline characteristics of the participants according to quartiles of cumMCMI.

Characteristics	Overall	cumMCMI				*P* value
		Q1	Q2	Q3	Q4	
Number	3,466	872	868	869	857	
Age, years (M, IQR)	57.00 (51.00, 63.00)	58.00 (51.00, 65.00)	57.00 (51.00, 63.00)	56.00 (49.00, 63.00)	57.00 (51.00, 63.00)	0.016
Gender *n* (%)						<0.001
Female	1,880 (54%)	310 (36%)	452 (52%)	520 (60%)	598 (70%)	
Male	1,586 (46%)	562 (64%)	416 (48%)	349 (40%)	259 (30%)	
Marital status *n* (%)						0.788
Non-married	451 (13%)	116 (13%)	120 (14%)	109 (13%)	106 (12%)	
Married	3,015 (87%)	756 (87%)	748 (86%)	760 (87%)	751 (88%)	
Education *n* (%)						0.056
College or above	81 (2.3%)	17 (1.9%)	18 (2.1%)	25 (2.9%)	21 (2.5%)	
Middle school	945 (27%)	237 (27%)	225 (26%)	270 (31%)	213 (25%)	
Elementary school or below	2,440 (70%)	618 (71%)	625 (72%)	574 (66%)	623 (73%)	
Residence *n* (%)						<0.001
Urban	462 (13%)	77 (8.8%)	106 (12%)	139 (16%)	140 (16%)	
Rural	3,004 (87%)	795 (91%)	762 (88%)	730 (84%)	717 (84%)	
Smoking *n* (%)						<0.001
Current smoker	1,052 (30%)	396 (45%)	281 (32%)	209 (24%)	166 (19%)	
Former smoker	248 (7.2%)	67 (7.7%)	54 (6.2%)	70 (8.1%)	57 (6.7%)	
Never smoked	2,166 (62%)	409 (47%)	533 (61%)	590 (68%)	634 (74%)	
Drinking *n* (%)						<0.001
Current drinker	1,112 (32%)	375 (43%)	287 (33%)	249 (29%)	201 (23%)	
Former drinker	255 (7.4%)	66 (7.6%)	63 (7.3%)	65 (7.5%)	61 (7.1%)	
Never drinker	2,099 (61%)	431 (49%)	518 (60%)	555 (64%)	595 (69%)	
Dyslipidemia *n* (%)						<0.001
No	3,186 (92%)	843 (97%)	818 (94%)	806 (93%)	719 (84%)	
Yes	280 (8.1%)	29 (3.3%)	50 (5.8%)	63 (7.2%)	138 (16%)	
Hypertension *n* (%)						<0.001
No	2,736 (79%)	776 (89%)	739 (85%)	671 (77%)	550 (64%)	
Yes	730 (21%)	96 (11%)	129 (15%)	198 (23%)	307 (36%)	
Diabetes *n* (%)						<0.001
No	3,275 (94%)	855 (98%)	840 (97%)	826 (95%)	754 (88%)	
Yes	191 (5.5%)	17 (1.9%)	28 (3.2%)	43 (4.9%)	103 (12%)	
Kidney disease *n* (%)						0.388
No	3,324 (96%)	828 (95%)	834 (96%)	839 (97%)	823 (96%)	
Yes	142 (4.1%)	44 (5.0%)	34 (3.9%)	30 (3.5%)	34 (4.0%)	
Liver disease *n* (%)						0.367
No	3,374 (97%)	842 (97%)	845 (97%)	850 (98%)	837 (98%)	
Yes	92 (2.7%)	30 (3.4%)	23 (2.6%)	19 (2.2%)	20 (2.3%)	
Overweight *n* (%)						<0.001
No	1,684 (49%)	740 (85%)	528 (61%)	315 (36%)	101 (12%)	
Yes	1,782 (51%)	132 (15%)	340 (39%)	554 (64%)	756 (88%)	
Depression *n* (%)						0.109
No	2,271 (66%)	554 (64%)	552 (64%)	590 (68%)	575 (67%)	
Yes	1,195 (34%)	318 (36%)	316 (36%)	279 (32%)	282 (33%)	
Antihypertensive drug *n* (%)						<0.001
No	2,967 (86%)	812 (93%)	790 (91%)	740 (85%)	625 (73%)	
Yes	499 (14%)	60 (6.9%)	78 (9.0%)	129 (15%)	232 (27%)	
Antidiabetic drug *n* (%)						<0.001
No	3,351 (97%)	861 (99%)	849 (98%)	849 (98%)	792 (92%)	
Yes	115 (3.3%)	11 (1.3%)	19 (2.2%)	20 (2.3%)	65 (7.6%)	
Lipid-lowering drug *n* (%)						<0.001
No	3,332 (96%)	858 (98%)	848 (98%)	842 (97%)	784 (91%)	
Yes	134 (3.9%)	14 (1.6%)	20 (2.3%)	27 (3.1%)	73 (8.5%)	
LDL-C mg/dL (*M*, IQR)	114.43 (93.56, 138.02)	106.70 (87.95, 126.42)	114.63 (95.10, 136.28)	120.23 (99.74, 145.75)	117.53 (92.78, 143.43)	<0.001
TC mg/dL (*M*, IQR)	190.98 (167.78, 216.11)	183.44 (163.15, 206.44)	186.34 (164.11, 209.54)	193.69 (170.49, 220.75)	200.65 (174.74, 226.16)	<0.001
HbA1C (%) (*M*, IQR)	5.10 (4.90, 5.40)	5.10 (4.80, 5.30)	5.10 (4.80, 5.30)	5.10 (4.80, 5.40)	5.30 (5.00, 5.70)	<0.001
cumMCMI (MCMI-years) (*M*, IQR)	8.62 (7.38, 9.97)	6.68 (6.15, 7.03)	8.03 (7.70, 8.35)	9.20 (8.91, 9.57)	11.11 (10.51, 12.00)	<0.001
Follow-up time (months) (*M*, IQR)	60.00 (59.00, 60.00)	60.00 (59.00, 60.00)	60.00 (59.00, 60.50)	60.00 (59.00, 60.00)	60.00 (30.50, 60.00)	<0.001
CVD *n* (%)						<0.001
No	2,816 (81%)	755 (87%)	723 (83%)	696 (80%)	642 (75%)	
Yes	650 (19%)	117 (13%)	145 (17%)	173 (20%)	215 (25%)	

For continuous non-normal variables, the median and interquartile range describe the data, and the Kruskal–Wallis's test provides the *P* value. For categorical variables, the rate is detailed, and the chi-square test provides the *P* value. cumMCMI, Quartiles: Q1 (cumMCMI < 7.38), Q2 (7.38 ≤ cumMCMI < 8.62), Q3 (8.62 ≤ cumMCMI < 9.97), Q4 (9.97 ≤ cumMCMI). *M*, IQR, median, interquartile range; LDL-C, low-density lipoprotein cholesterol; TC, total cholesterol; HbA1C, hemoglobin A1C; cumMCMI, cumulative modified cardiometabolic index; CVD, cardiovascular disease.

### Link between CVD and cumMCMI

**Figure 2:**
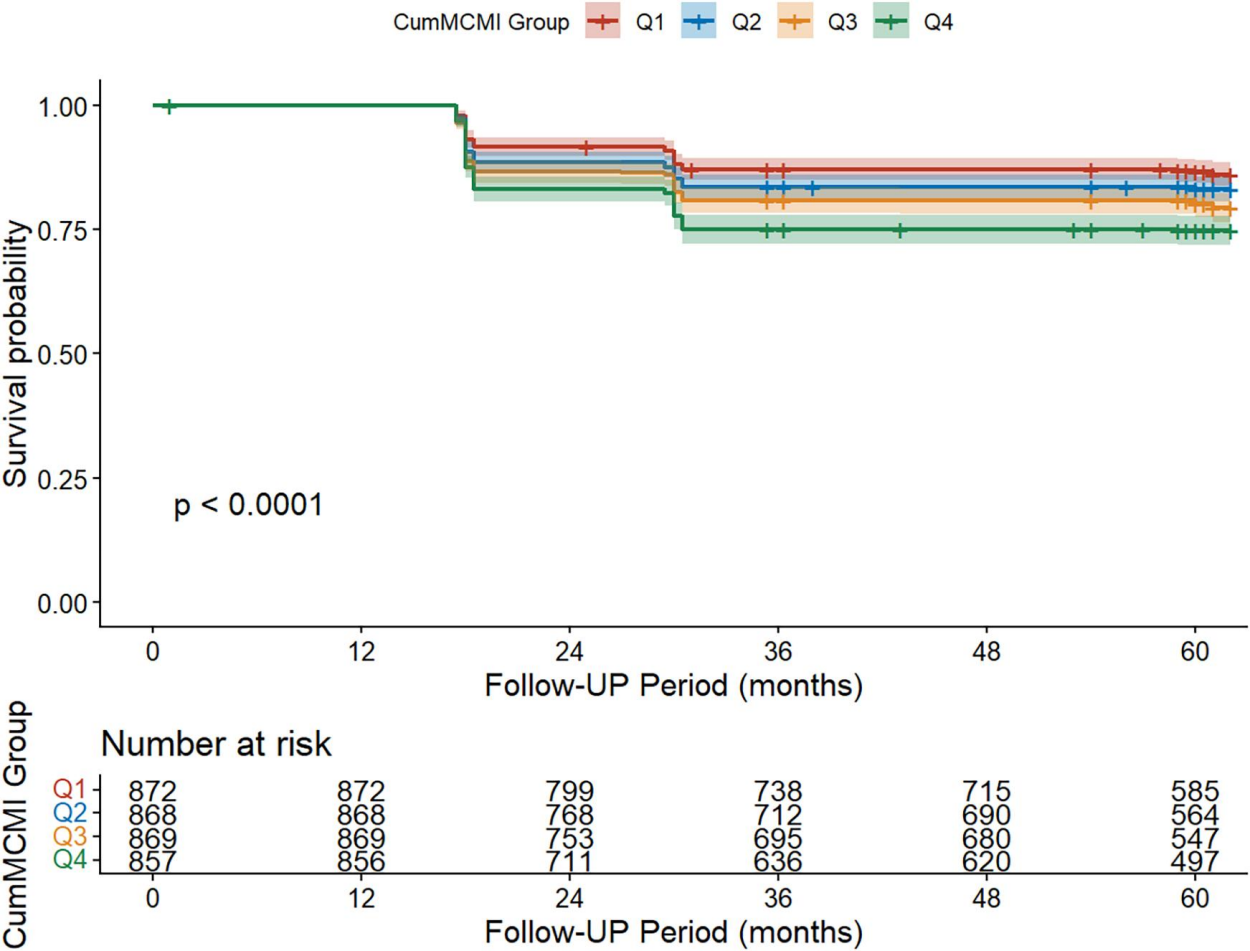
Kaplan–Meier survival curve between cumMCMI and CVD. cumMCMI, Quartiles: Q1 (cumMCMI < 7.38), Q2 (7.38 ≤ cumMCMI < 8.62), Q3 (8.62 ≤ cumMCMI < 9.97), Q4 (9.97 ≤ cumMCMI). cumMCMI, cumulative modified cardiometabolic index; CVD, cardiovascular disease.

**Figure 3:**
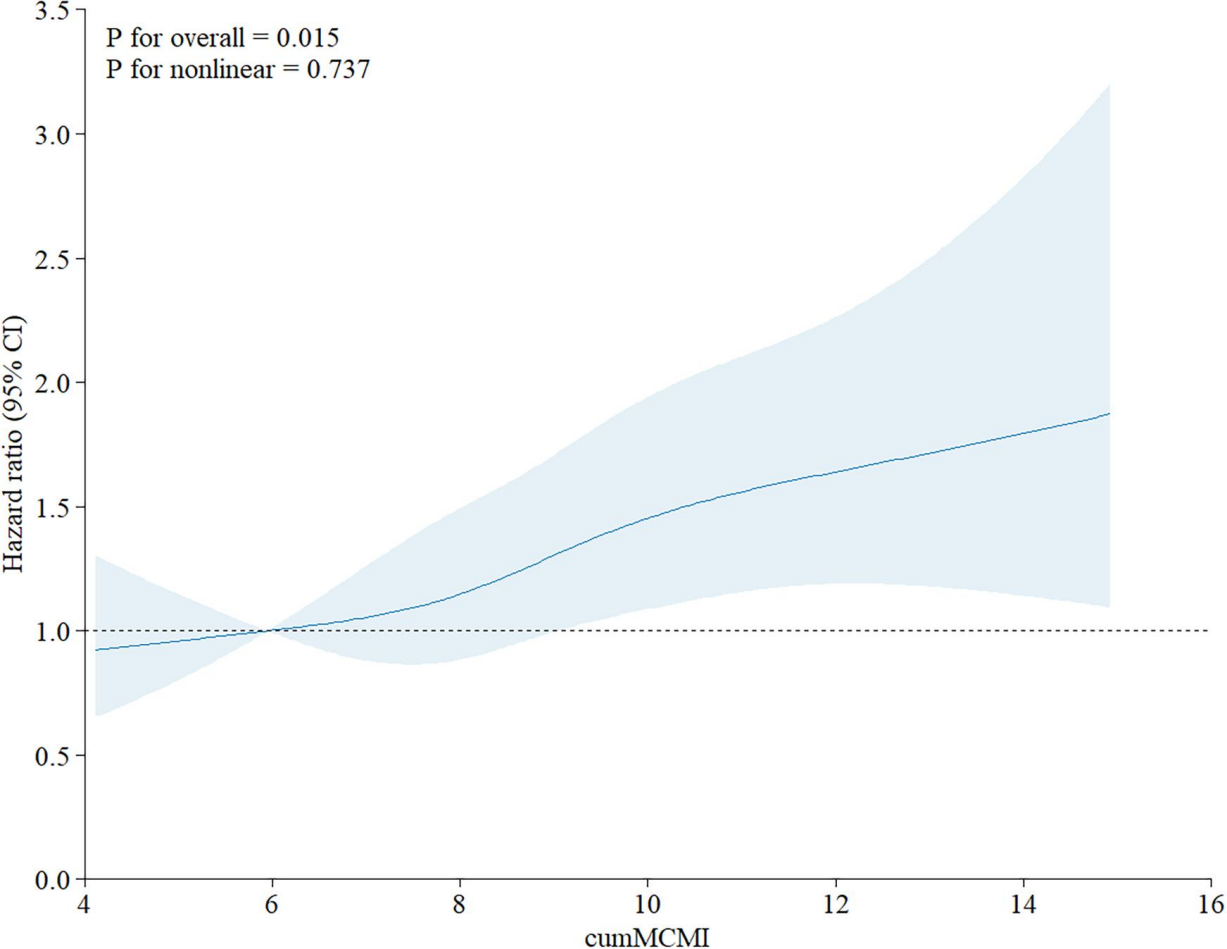
Dose-response association between cumMCMI and CVD. The solid line represents the HR, and the shadow represents the 95% CI. The *P* value for overall association was 0.015, and for non-linearity was 0.737. All covariates were adjusted in this model. cumMCMI, cumulative modified cardiometabolic index.

By including cumMCMI as a continuous variable in the unadjusted Cox model, it was found that each unit increase was linked with a higher risk of CVD (HR = 1.14, 95% CI: 1.10–1.18, *P* < 0.001). After adjusting for various factors, the association weakened but stayed significant (Model 3: HR = 1.09, 95% CI: 1.03–1.14, *P* = 0.002). The unadjusted model showed statistically significant HR of 1.55 for Q3 and 2.00 for Q4, whereas the HR for Q2 was 1.27 and lacked statistical significance. The trend continued in the multivariable-adjusted model, with Q2 remaining non-significant, while Q3 and Q4 had significant HRs. The HR for Q4 decreased from 2.00 to 1.51 in Model 3, demonstrating a clear dose-response relationship (*P* for trend < 0.05) (Table 2). The incidence of CVD was significantly higher among those in the uppermost quartile, as evidenced by the log-rank test (*P* < 0.001) and the corresponding Kaplan–Meier curves (Figure 2). The dose-response relationship between cumMCMI and CVD risk was characterized by using RCS to account for potential linearity. Even after accounting for potential covariates, a significant positive association between cumMCMI and CVD risk persisted (*P* = 0.015), with no evidence of a non-linear departure (*P* for nonlinearity = 0.737). Data from the Cox regression model (HR = 1.09, 95% CI: 1.03–1.14) further support a linear escalation in CVD susceptibility per unit increase of cumMCMI, confirming the absence of a discernible threshold effect (Figure 3).

**Table 2:** Cox regression analysis of the link between cumMCMI and cardiovascular disease across various models.

Variable	Crude model HR (95%CI)	*P* value	Model 1 HR (95%CI)	*P* value	Model 2 HR (95%CI)	*P* value	Model 3 HR (95%CI)	*P* value
cumMCMI per unit	1.14 (1.10, 1.18)	<0.001	1.13 (1.09, 1.18)	<0.001	1.12 (1.07, 1.18)	<0.001	1.09 (1.03, 1.14)	0.002
Quartiles of cumMCMI								
Q1	ref		ref		ref		ref	
Q2	1.27 (0.998, 1.62)	0.052	1.26 (0.99, 1.61)	0.065	1.21 (0.95, 1.55)	0.128	1.19 (0.93, 1.53)	0.167
Q3	1.55 (1.22, 1.95)	<0.001	1.52 (1.19, 1.93)	<0.001	1.41 (1.11, 1.80)	0.006	1.35 (1.04, 1.75)	0.022
Q4	2.00 (1.60, 2.50)	<0.001	1.92 (1.52, 2.43)	<0.001	1.75 (1.37, 2.23)	<0.001	1.51 (1.13, 2.00)	0.005
*P* for trend		<0.001		<0.001		<0.001		0.005

HR, hazard ratio; 95% CI, 95% confidence interval; cumMCMI, Quartiles: Q1 (cumMCMI < 7.38), Q2 (7.38 ≤ cumMCMI < 8.62), Q3 (8.62 ≤ cumMCMI < 9.97), Q4 (9.97 ≤ cumMCMI). Multiple models: crude model: no covariable was adjusted; Model 1: adjusted for age, gender, marital status, residence, education; Model 2: adjusted for Model 1 + smoking status, drinking status, LDL-C, TC, HbA1C; Model 3: adjusted for Model 2 + hyperlipidemia, diabetes, kidney disease, liver disease, overweight, depression, antihypertensive drug, antidiabetic drug, lipid-lowering drug. LDL-C, low-density lipoprotein cholesterol; TC, total cholesterol; HbA1C, hemoglobin A1C; cumMCMI, cumulative modified cardiometabolic index.

**Table 3:** Subgroup analysis of cumMCMI link to cardiovascular disease incidence.

Subgroups	Event/Total (%)	HR (95% CI)	*P* value	*P* for interaction
Overall	3,466	1.15 (1.05, 1.25)	0.003	
Age, years				0.093
<45	94 (2.7)	2.86 (1.31, 6.26)	0.009	
≥45 and <60	1,988 (57.4)	1.14 (1.01, 1.30)	0.036	
≥60	1,384 (39.9)	1.11 (0.97, 1.27)	0.131	
Gender				0.068
Female	1,880 (54.2)	1.07 (0.95, 1.20)	0.276	
Male	1,586 (45.8)	1.28 (1.11, 1.48)	0.001	
Residence				0.121
Urban	462 (13.3)	0.96 (0.74, 1.23)	0.739	
Rural	3,004 (86.7)	1.19 (1.08, 1.31)	0.001	
Smoking				0.552
Current smoker	1,052 (30.4)	1.31 (1.10, 1.57)	0.003	
Former smoker	248 (7.2)	1.04 (0.73, 1.48)	0.83	
Never smoker	2,166 (62.5)	1.12 (1.00, 1.25)	0.053	
Drinking				0.112
Current drinker	1,112 (32.1)	1.37 (1.15, 1.62)	<0.001	
Former drinker	255 (7.4)	1.01 (0.75, 1.36)	0.944	
Never drinker	2,099 (60.6)	1.09 (0.97, 1.22)	0.154	
Marital status				0.032
Non-married	451 (13.0)	1.03 (0.80, 1.32)	0.816	
Married	3,015 (87.0)	1.17 (1.06, 1.29)	0.002	
Education				0.026
College or above	81 (2.3)	0.57 (0.28, 1.17)	0.125	
Middle school	945 (27.3)	1.21 (0.99, 1.48)	0.064	
Elementary school or below	2,440 (70.4)	1.15 (1.04, 1.28)	0.007	
Diabetes				0.703
No	3,275 (94.5)	1.16 (1.05, 1.27)	0.002	
Yes	191 (5.5)	1.01 (0.68, 1.50)	0.95	
Kidney disease				0.385
No	3,324 (95.9)	1.14 (1.04, 1.25)	0.005	
Yes	142 (4.1)	1.43 (0.91, 2.27)	0.123	
Liver disease				0.336
No	3,374 (97.3)	1.15 (1.04, 1.26)	0.004	
Yes	92 (2.7)	0.99 (0.56, 1.73)	0.958	
Dyslipidemia				0.724
No	3,186 (91.9)	1.15 (1.05, 1.27)	0.004	
Yes	280 (8.1)	1.13 (0.86, 1.48)	0.394	
Hypertension				0.511
No	2,736 (78.9)	1.11 (1.00, 1.23)	0.049	
Yes	730 (21.1)	1.28 (1.06, 1.55)	0.012	
Overweight				0.378
No	1,684 (48.6)	1.17 (1.02, 1.34)	0.022	
Yes	1,782 (51.4)	1.11 (0.98, 1.26)	0.09	
Depression				0.876
No	2,271 (65.5)	1.14 (1.01, 1.29)	0.029	
Yes	1,195 (34.5)	1.17 (1.02, 1.35)	0.028	
Antihypertensive drug				0.209
No	2,967 (85.6)	1.12 (1.01, 1.23)	0.027	
Yes	499 (14.4)	1.35 (1.05, 1.74)	0.02	
Antidiabetic drug				0.75
No	3,351 (96.7)	1.15 (1.05, 1.27)	0.003	
Yes	115 (3.3)	1.02 (0.60, 1.73)	0.939	
Lipid-lowering drug				0.982
No	3,332 (96.1)	1.15 (1.05, 1.27)	0.003	
Yes	134 (3.9)	1.08 (0.72, 1.62)	0.70	

HR, hazard ratio; 95% CI, 95% confidence interval. The model was adjusted for age, gender, marital status, residence, education, smoking status, drinking status, LDL-C, TC, HbA1C, hypertension, hyperlipidemia, diabetes, kidney disease, liver disease, overweight, depression, antihypertensive drug, antidiabetic drug, lipid-lowering drug. LDL-C, low-density lipoprotein cholesterol; TC, total cholesterol; HbA1C, hemoglobin A1C; cumMCMI, cumulative modified cardiometabolic index.

### Subgroup analysis

Within the total study cohort, cumMCMI emerged as a robust predictor of the outcome (*P* = 0.003), yielding an estimated effect size of 1.15 (95% CI: 1.05–1.25). Subgroup analysis showed significant differences by marital status (*P* for interaction = 0.032), with only married individuals showing a significant effect, and by educational level (*P* for interaction = 0.026), where cumMCMI was higher in those with elementary school or below. While not reaching strict statistical significance, the interaction tests for age (*P* = 0.093) and sex (*P* = 0.068) points toward a slightly more robust association in men and younger individuals (aged < 45). No significant differences were found for residence, smoking status, drinking status, kidney disease, liver disease, dyslipidemia, hypertension, diabetes, overweight, depression, antihypertensive drug, antidiabetic drug, and lipid-lowering drug, as their *P* for interaction values were all >0.05 (Table 3).

### Sensitivity analysis

A series of three sensitivity analyses was conducted to verify the stability of our core results. Participants were initially split into high and low exposure groups based on the median cumMCMI, and then an inverse probability of treatment weighting (IPTW) Poisson regression model was applied. A notably increased CVD risk was observed in the high-exposure group (RR = 1.30, 95% CI: 1.10–1.53, *P* = 0.002), and the corresponding *E*-value was 1.92. The exposure cutoff was raised to the highest quartile, yielding an RR estimate of 1.27 with a 95% CI of 1.04–1.55, significantly above the null (*P* = 0.02). To tackle potential model-specification bias, we used a weighted logistic regression model. The odds ratio was 1.18, with a 95% CI of 1.06–1.30 (*P* = 0.002), and the corresponding *E*-value was 1.63. These sensitivity analyses yielded materially similar results (Table 4).

**Table 4:** Results of sensitivity analysis.

Sensitivity analysis	Exposure definition	Effect size (95% CI)	*P* value	*E*-value (95% CI lower bound)
Analysis 1	Median cut-off (high vs. low)	1.30 (1.10, 1.53)	0.002	1.92 (1.44)
Analysis 2	Upper quartile (Q4 vs. Q1–Q3)	1.27 (1.04, 1.55)	0.02	–
Analysis 3	Median cut-off (logistic regression)	1.18 (1.06, 1.30)	0.002	1.63 (1.32)

Analysis 1 and Analysis 2 both utilize Poisson regression but differ in their cutoff points, using the median and upper quartile, respectively. Analysis 1 and 3 are based on the same exposure definition (median cut-off) but employ different models (Poisson vs. logistic regression). The *E*-value calculations are based on the corresponding effect sizes and their confidence intervals.

## Discussion

The CHARLS prospective study demonstrated a strong positive link between cumMCMI and the risk of developing new CVD, which persisted even after adjusting for all potential covariates in the multivariable Cox regression. Stability was noted when considering cumMCMI as a continuous variable and by quartiles. The dose-response pattern between cumMCMI and CVD incidence appeared predominantly linear in the RCS model. The absence of a threshold or plateau effect was further substantiated by the linearity test (*P* for nonlinearity = 0.737). Educational attainment and marital status showed interaction *P* values less than 0.05 in subgroup analyses; this hints at a possible interaction where education and marital status moderate the impact of cumMCMI on CVD development. However, due to small sample sizes for highly educated (2.3%) and unmarried (13%) participants, this result should be cautiously interpreted.

As shown in Table 1, the rise in cumMCMI corresponded with an increase in female participants from 36% in Q1 to 70% in Q4, while the percentages of individuals who currently smoke or drink decreased significantly, with smokers reducing from 45% to 19% and drinkers from 43% to 23%. This distribution pattern may be related to differences in sex composition, behavioral modification, residual confounding, or survivor/selection effects. Therefore, the association between cumMCMI and incident CVD was interpreted mainly based on the multivariable-adjusted Cox models. The HR for cumMCMI decreased after multivariable adjustment but remained statistically significant, suggesting that the observed association was not fully explained by the measured covariates. The study's quartile analysis showed that, compared to Q1, the Q2 group's *P* value was above 0.05, while Q3 and Q4 had significantly higher HRs with a trend test *P* value under 0.05. However, RCS results (*P* nonlinear = 0.737) indicated a linear relationship between cumMCMI and CVD risk without a threshold effect. The lack of significance in Q2 is likely due to low statistical power from a small effect size, not the absence of an effect. Overall, a linear increase was observed, supporting a consistent dose-response link between cumMCMI and CVD risk.

Subgroup analysis revealed significant interactions between educational level, marital status, and cumMCMI on CVD risk (*P* < 0.05), suggesting that cumMCMI's impact varies with education and marital status. Table 1 shows that Q1 had more well-educated individuals, while Q4 had more with lower education, aligning with previous studies highlighting education as crucial for cardiovascular health (23). Only 2.3% of the sample had higher education, raising concerns about the study's statistical power and effect stability. Marital status influenced the effect of cumMCMI: married individuals saw a 17% risk increase per unit (*P* < 0.05, 95% CI: 1.06–1.29), while unmarried individuals showed no significant association (HR: 1.03, 95% CI: 0.80–1.32). Unmarried individuals made up just 13.0% of the group, suggesting the interaction might be due to sample size imbalance rather than a biological effect. Additionally, marital status could indicate socioeconomic status and social support, linking it to unmeasured factors like financial stress and healthcare access (24), which may differently impact cumMCMI effects across marital groups.

Risk factors like dyslipidemia (25–27), impaired glucose tolerance (28, 29), and central obesity (30, 31) can cause excess lipids to build up in unusual areas, such as epicardial adipose tissue (32) around the heart. This buildup triggers oxidative stress (33) and inflammation, thereby activating signaling pathways such as NF-κB pathway (34) and advancing CVD. Previous studies have primarily measured individual indicators such as LDL (7), FBG (10, 35, 36), and TG (12) in isolation. When evaluating the relative efficacy of the TyG index vs. the MCMI for predicting incident T2DM, the MCMI consistently yielded a higher predictive utility (15). The CMI, which does not account for abnormal blood glucose levels, has demonstrated good predictive performance in chronic metabolic diseases such as CVD (37–39), whereas cumMCMI integrates the long-term intensity and duration of exposure to indicators such as dyslipidemia, impaired glucose tolerance, and central obesity, thus effectively encapsulating the long-term, aggregate impact of metabolic disturbances on cardiovascular health. Previous studies have provided important evidence regarding MCMI and cardiometabolic outcomes. Pan et al. evaluated baseline MCMI in relation to a broad range of chronic diseases and compared its predictive performance with the original CMI (16). Chen et al. examined baseline MCMI in relation to cardiometabolic multimorbidity using CHARLS and English Longitudinal Study of Ageing (ELSA) (17), whereas Wang et al. investigated baseline MCMI and CVD outcomes in individuals with early CKM syndrome (18). In contrast to these baseline-exposure studies, the present analysis focused on cumMCMI derived from repeated CHARLS biomarker measurements and specifically evaluated its association with incident CVD in a broader middle-aged and older population. Therefore, our findings extend previous CHARLS-based evidence by suggesting that the duration and accumulation of cardiometabolic burden may also be relevant to future CVD risk. Nevertheless, our findings should not be interpreted as demonstrating that cumMCMI is definitively superior to other cumulative metabolic indices, because direct head-to-head comparisons and the establishment of standardized clinical cutoffs were beyond the scope of the present study. In addition, although composite indices may improve the integration of metabolic information, this does not necessarily translate into a clear clinical advantage. Our study was not designed to directly compare cumMCMI with other established glucose-integrated indices, such as TyG, metabolic score for insulin resistance (METS-IR), homeostasis model assessment-insulin resistance (HOMA-IR), or the AIP + FBG combination. Therefore, the incremental clinical value of cumMCMI remains to be further validated. Nevertheless, because cumMCMI is derived from routinely available anthropometric and laboratory parameters, its potential applicability in population-based cardiometabolic risk assessment warrants further investigation.

This study has several strengths: it established a linear dose-response relationship between cumMCMI and CVD using RCS, balanced confounder distribution with an IPTW generalized linear model, and confirmed the robustness of findings through multiple sensitivity analyses with varied exposure cutoffs and models. Nonetheless, our study has certain limitations. First, the exclusion of participants with incomplete interview or biomarker data may have introduced some degree of selection bias. Excluded individuals may have differed from included participants in terms of health status or socioeconomic characteristics. Therefore, the generalizability of our findings to the broader CHARLS population should be interpreted with caution. Second, although we calculated an *E*-value to evaluate unmeasured confounding, the *E*-value was 1.92 (95% CI lower limit: 1.44) on the RR scale and 1.63 (95% CI lower limit: 1.32) on the OR scale, suggesting that relatively strong unmeasured confounding would be required to fully explain the observed association. However, this does not exclude the possibility of such confounding, and unmeasured confounding cannot be completely ruled out. Third, because the CHARLS database only provides cause-of-death data in Wave 2, this study cannot differentiate between non-fatal CVD events and CVD-related deaths, nor fully consider the effect of competing risks like non-cardiovascular deaths. Future studies with access to cause-of-death data should validate findings using a competing risks model.

## Conclusion

In summary, cumMCMI was independently and positively associated with CVD. As it combines data on long-term lipid levels, fasting glucose, and central obesity, cumMCMI may have potential value for CVD stratification and early warning. Educational attainment and marital status might influence this association, but further validation is needed. Future studies should confirm these findings and further assess the predictive value and clinical applicability of cumMCMI.

## Data Availability

Publicly available datasets were analyzed in this study. Researchers can apply to download the data at http://charls.pku.edu.cn/.
